# Comparison between Limbal and Pars Plana Approaches Using Microincision Vitrectomy for Removal of Congenital Cataracts with Primary Intraocular Lens Implantation

**DOI:** 10.1155/2016/8951053

**Published:** 2016-05-30

**Authors:** Xin Liu, Tianyu Zheng, Xingtao Zhou, Yi Lu, Peng Zhou, Fan Fan, Yi Luo

**Affiliations:** ^1^Department of Ophthalmology, Eye and ENT Hospital of Fudan University, Shanghai 200031, China; ^2^Key Laboratory of Myopia of State Health Ministry, Shanghai 200031, China; ^3^Key Laboratory of Visual Impairment and Restoration of Shanghai, Shanghai 200031, China; ^4^Department of Ophthalmology, ParkwayHealth Hong Qiao Medical Center, Shanghai 200033, China

## Abstract

*Purpose*. To compare the surgical outcomes of limbal versus pars plana vitrectomy using the 23-gauge microincision system for removal of congenital cataracts with primary intraocular lens implantation.* Methods*. We retrospectively reviewed all eyes that underwent cataract removal through limbal or pars plana incision. Main outcome measures included visual outcomes and complications.* Results*. We included 40 eyes (26 patients) in the limbal group and 41 eyes (30 patients) in the pars plana group. The mean age was 46 months. There was no significant difference in best-corrected visual acuity between the two groups (*P* = 0.64). Significantly, more eyes had at least one intraoperative complication in the limbal group than in the pars plana group (*P* = 0.03) that were mainly distributed at 1.5–3 years of age (*P* = 0.01). The most common intraoperative complications were iris aspiration, iris prolapse, and iris injury. More eyes in the limbal group had postoperative complications and required additional intraocular surgery, but the difference was not significant (*P* = 0.19).* Conclusions*. The visual results were encouraging in both approaches. We recommend the pars plana approach for lower incidence of complications. The limbal approach should be reserved for children older than 3 years of age and caution should be exercised to minimize iris disturbance.

## 1. Introduction

Congenital cataract is the leading cause of treatable blindness in children worldwide [1–3]. Surgical management of congenital cataracts is a challenging task because of the elastic capsule, high pressure of the posterior chamber, and elevated inflammatory reaction due to irritation of the iris. In the mid-1970s, the utilization of vitreous suction-cutting devices to perform primary posterior capsulotomy and anterior vitrectomy revolutionized pediatric cataract surgery [4, 5]. The introduction of microincision vitrectomy instruments further minimized surgically induced trauma and inflammation and therefore hastened postoperative recuperation and enabled immediate optical correction and amblyopic treatment [6, 7].

There are two approaches to performing primary capsulotomy and anterior vitrectomy using the microincision vitrectomy system for the management of congenital cataracts: through the limbus [8–11] and through the pars plana [12–14]. When managing infantile congenital cataract without primary intraocular lens (IOL) implantation, the pars plana approach has the advantages of a more sufficient lensectomy and anterior vitrectomy, reduced surgical trauma, and postoperative inflammation, and therefore it is recommended by most surgeons [12–14]. When managing older children with primary IOL implantation, another limbal incision is required and the surgical procedure is complex. Some studies have advocated the limbal approach for the advantages of more precise manipulations under direct vision, a more stable anterior chamber, and a shorter learning curve. We also used the limbal approach for pediatric congenital cataract, but the problem of increased irritation to the iris resulting in elevated inflammatory reactions could not be ignored [8–10]. Despite the advantages and disadvantages of the two approaches presented in past studies, including ours, the current management of congenital cataract uses the microincision vitrectomy system with only a single one of the above-mentioned approaches. The question regarding which approach is more appropriate for the management of cataracts in older children with primary IOL implantation remains unaddressed. There is no report comparing the outcomes of these two approaches using microincision vitrectomy. We therefore conducted a retrospective study to compare the visual outcomes and adverse events of the limbal versus the pars plana approaches.

## 2. Materials and Methods

### 2.1. Patients

We retrospectively reviewed all patients with congenital cataracts who underwent cataract removal through a limbal or a pars plana incision using a 23-gauge vitrectomy system with primary IOL implantation between August 2009 and August 2013 at the Eye and ENT Hospital of Fudan University. Patients who underwent the cataract surgery through the limbal approach were included in the limbal group. Children who had cataract removal using a pars plana approach were included in the pars plana group. The follow-up period was at least 2 years. Exclusion criteria were traumatic, subluxated, or complicated cataracts and evidence of any ocular or systemic anomalies. Informed consent was obtained from the parents of all participating children. This study was carried out with the approval of the institutional review board of the Eye and ENT Hospital of Fudan University and in accordance with the Declaration of Helsinki.

Each patient underwent a detailed preoperative evaluation under sedation by chloral hydrate including intraocular pressure (IOP) measurement, slit-lamp examination, corneal endothelial cell density (ECD) calculation, and B ultrasound. Axial length was measured by an ultrasonic A-scan (Nidek US-800; Nidek, Fremont, CA, USA). The IOL power was based on the SRK-T formula targeting hyperopia.

### 2.2. Surgical Procedures

All surgeries were performed under general anesthesia by one surgeon (Y. Luo) with the Millennium Microsurgical System (Alcon, Fort Worth, TX, USA) and the 23-gauge microincision vitrectomy system.

**Figure 1:**
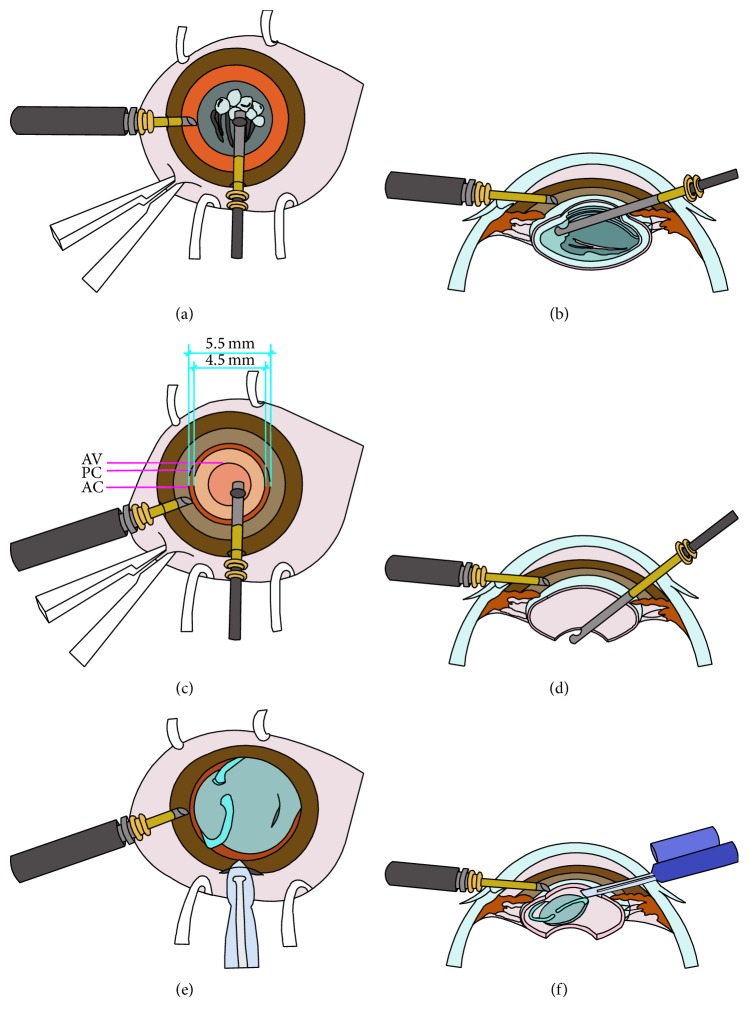
(a) Lensectomy was performed after a central anterior capsulotomy of 5.5 mm diameter using a 23-gauge vitrectomy cutter via a limbal incision. A limbal port incision was made for infusion to maintain the anterior chamber. The eye was positioned using a pair of forceps. (b) Cross-sectional diagram shows the lensectomy procedure. (c) Anterior vitrectomy after a posterior capsulotomy of 4.5 mm diameter. (d) Cross-sectional diagram shows the anterior vitrectomy procedure. (e) The 12 o'clock limbal incision was enlarged and a one-piece foldable IOL was implanted into the capsule bag. (f) Cross-sectional diagram shows the IOL implantation procedure (AV = anterior vitrectomy; AC = anterior capsulotomy; PC = posterior capsulotomy).

**Figure 2:**
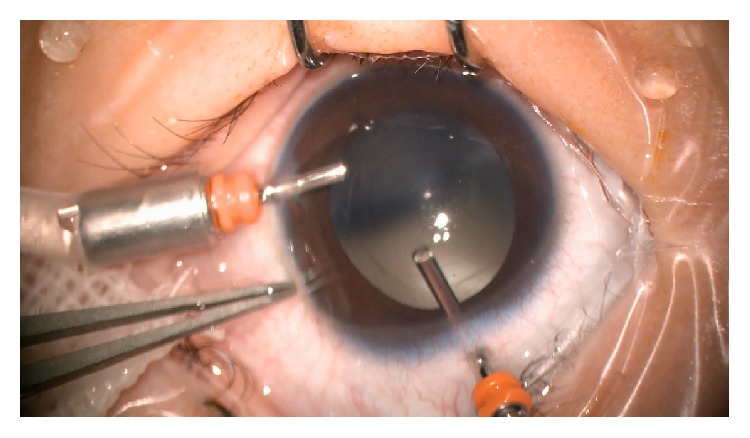
Intraoperative video frame shows the operated eye of the limbal group after anterior capsulotomy, after lensectomy, after posterior capsulotomy, and during anterior vitrectomy.

For children in the limbal group, cataract surgery was performed through two limbal incisions made by a 23-gauge trocar with a microcannula. A 23-gauge infusion cannula was inserted through the 4 or 8 o'clock limbal incision to maintain the anterior chamber with balanced salt solution (BSS; Alcon). A cutting tip of the 23-gauge vitrectomy instrument was introduced through an incision at the 12 o'clock position. A central anterior capsulotomy of 5.0–5.5 mm diameter was created using the vitrector. Lens material was removed at a cutting rate of 600 cuts per minute and a maximum suction pressure of 400 mmHg (Figures 1(a) and 1(b)). A posterior capsulotomy of 4.0–4.5 mm diameter was created followed by a limited anterior vitrectomy (Figures 1(c), 1(d), and 2). The microcannula at the 12 o'clock incision was then removed without suturing (see video 1 in Supplementary Material available online at http://dx.doi.org/10.1155/2016/8951053).

**Figure 3:**
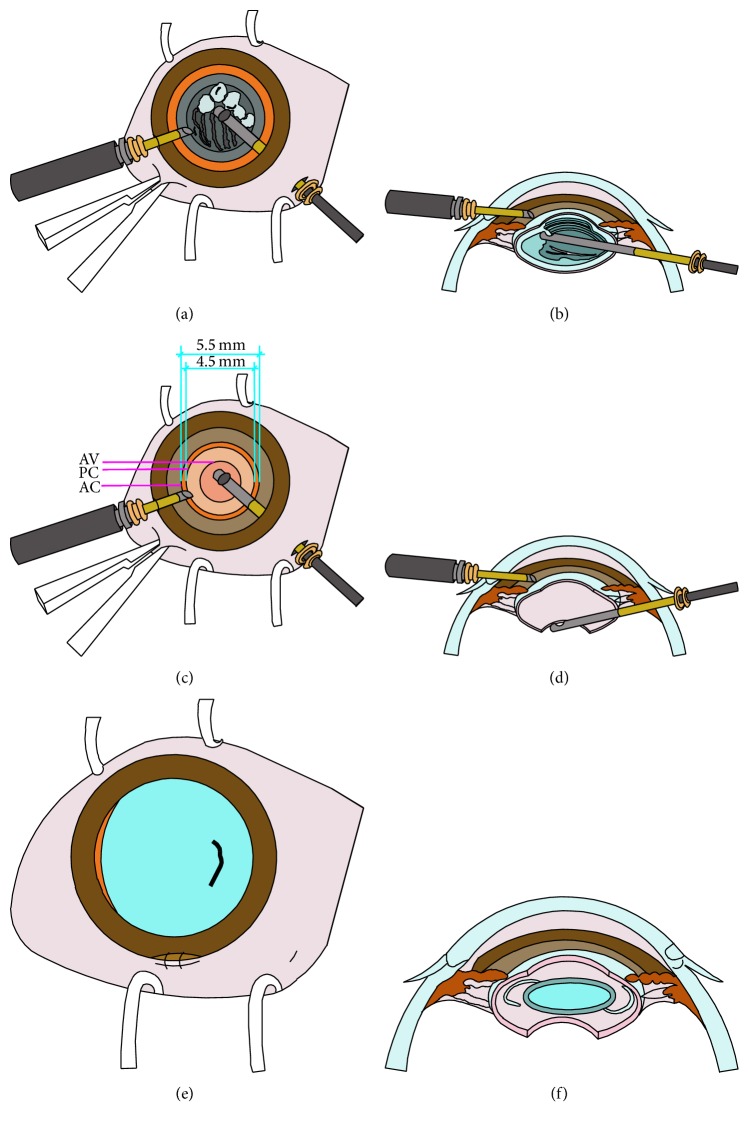
(a) Lensectomy was performed after a central anterior capsulotomy using a 23-gauge vitrectomy cutter via a pars plana incision, which was 2.5 mm posterior to the limbus. (b) Cross-sectional diagram shows the lensectomy procedure. (c) Anterior vitrectomy after a posterior capsulotomy of 4.5 mm diameter. (d) Cross-sectional diagram shows the anterior vitrectomy procedure. (e) Status at the end of surgery. Another 2.6 mm limbal incision was made for IOL implantation and was closed with 2 sutures at the end of the surgery. (f) Cross-sectional diagram shows the status at the end of surgery.

**Figure 4:**
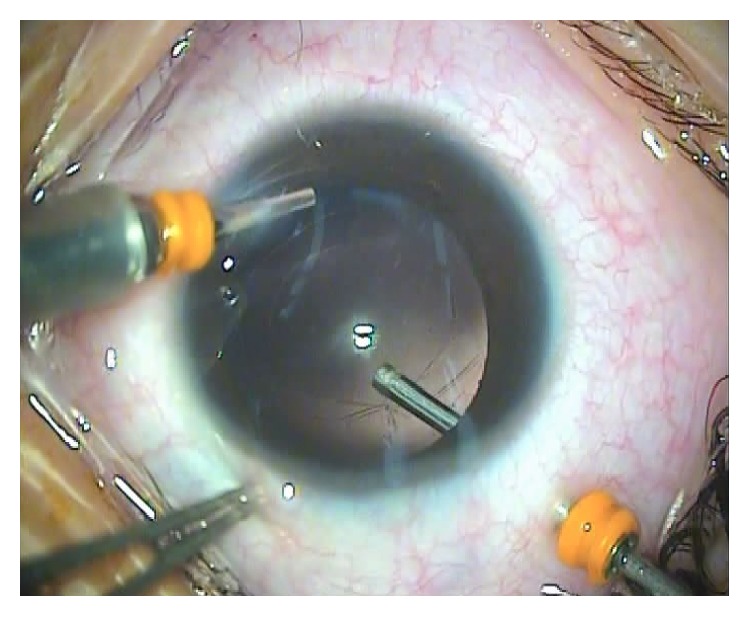
Intraoperative video frame shows the operated eye of the pars plana group B after anterior capsulotomy, after lensectomy, after posterior capsulotomy, and during anterior vitrectomy.

For children in the pars plana group, cataract removal was performed as previously described [14]. Briefly, a 23-gauge infusion cannula was inserted through a limbal port incision to maintain the anterior chamber with BSS. A pars plana incision was made at the 10 o'clock position and a 23-gauge vitrectomy cutter with a microcannula was introduced. Because the pars plana is not well developed in young children, the options for the sclerotomy sites differ according to each patient's age [15]. In our series, a sclerotomy site was chosen 2.5 mm posterior to the limbus in patients aged 1.5 to 3 years and 3.0 mm in those aged 3 to 6 years. A central anterior capsulotomy, lensectomy, posterior capsulotomy, and limited anterior vitrectomy were performed using the cutter at the same setting described above (Figures 3(a)–3(d) and 4). The microcannula at the pars plana incision was then removed without suturing (Supplementary Material, video 2).

All eyes had primary IOL implantation. For eyes in the limbal group, the 12 o'clock limbal incision was enlarged to 2.6 mm. For eyes in the pars plana group, another 2.6 mm limbal incision was made at the 12 o'clock position. The limbal incisions were made through the conjunctiva without a conjunctival flap. After the ophthalmic viscosurgical device (OVD) (DisCoVisc; Alcon) was injected, a one-piece foldable IOL (AcrySof SA60AT; Alcon) was implanted into the capsular bag (Figures 1(e) and 1(f)). The limbal incision was closed with one or two 10-0 nylon sutures (Ethilon 9033; Allmedtech, Beverley Hills, CA, USA), and the corneal stroma at the limbal side port was hydrated with BSS after removal of the infusion cannula (Figures 3(e) and 3(f); Supplementary Material, videos 1 and 2).

Postoperatively, topical eyedrops containing 0.3% tobramycin and 0.1% dexamethasone were used three times daily for 2 weeks, and pranoprofen ophthalmic solution was used three times daily for 1 month. All patients underwent refraction by retinoscopy 3–5 days after surgery. Spectacles or contact lenses were prescribed to correct any residual refractive error in pseudophakic eyes. In unilateral cases, occlusion therapy for the fellow eye was prescribed for 4 to 8 hours every day. Amblyopia treatment was initiated and efforts were made to encourage development of binocular function.

### 2.3. Follow-Up

The patients were examined postoperatively at 1 day, 1 week, and 1 month and at intervals of 3 months thereafter. Each examination included a complete ophthalmological evaluation and update of optical correction and monitoring of amblyopia treatment.

### 2.4. Main Outcome Measures

Main outcome measures included visual acuity, postoperative refraction, and complications. Definitions for intraoperative complications were as follows: iris prolapse, extrusion of the iris through the operative wound during surgery; iris aspiration, inadvertent aspiration of iris tissue by the vitrector with or without subsequent injury to the iris during surgery; iris injury, permanent structural change to the iris occurring during surgery; lens fragments in the vitreous, known loss of lens fragments into the vitreous requiring a pars plana approach for removal; and tear of the posterior capsule, tear of the posterior capsule to the equator of the lens during the procedure.

### 2.5. Data Analyses

Best-corrected visual acuity (BCVA) data were converted to the logarithm of the minimum angle of resolution (logMAR) scores for statistical analysis. Categorical variables were compared between groups using Fisher's exact test. All tests were two-tailed. Numerical variables were compared using a *t*-test or Wilcoxon rank sum test for independent samples and a paired Student's *t*-test for paired samples. A *P* value of < 0.05 was considered statistically significant. All analyses were conducted using SPSS, version 16.0 (SPSS Inc., Chicago, IL, USA).

## 3. Results

This retrospective study consisted of 40 eyes of 26 children who underwent cataract surgery through a limbal approach (limbal group), and 41 eyes of 30 children had removal of lens material using a pars plana approach (pars plana group). Baseline patient demographics were comparable between the groups (Table 1). The follow-up period was significantly longer in the pars plana group compared with the limbal group (*P* < 0.001).

### 3.1. Visual Results

Preoperatively, 41 patients (56 eyes, 69.1%) could cooperate with measurement of visual acuity. At last follow-up, all children could cooperate. The mean logMAR BCVA was significantly improved after cataract surgery (*P* < 0.001). Twenty-six eyes (65.0%) in the limbal group and 25 eyes (61.0%) in the pars plana group had a logMAR BCVA of 0.3 or less. The significantly higher mean refraction at last follow-up in the limbal group (*P* = 0.001) was associated with the shorter follow-up period resulting in younger ages at last follow-ups (Table 2).

### 3.2. Intraoperative Complications

There were significantly more eyes with one or more intraoperative complications in the limbal group than in the pars plana group (*P* = 0.032) (Table 3). Iris aspiration usually occurred during the lensectomy process. There were significantly more eyes with iris aspiration in the limbal group than the pars plana group (*P* = 0.005). Ten of the 13 incidences in the limbal group and the three in the pars plana group of iris aspiration resulted in no known adverse sequelae because of immediate release of the iris. The other three are detailed under iris injury.

Iris prolapse usually occurred during limbal incision made for IOL placement or during IOL implantation. Thirteen cases were associated with no known adverse sequelae. One eye in the limbal group resulted in iris incarceration in the 12 o'clock limbal incision the first day after surgery and required surgery to reposition the iris. In the other case of iris injury, and following iris aspiration, a 1*∗*1 mm defect of the iris resulted in a pupil that was not round. The other three cases were minor defects of less than 0.5 mm in diameter.

Tear of the posterior capsule occurred during posterior capsulotomy in 8 eyes. A three-piece IOL (AR40e; AMO, Santa Ana, CA, USA) was implanted in the sulcus.

Lens fragments in the vitreous occurred in one eye of the limbal group and necessitated a pars plana approach to removing it at the time of original surgery.

Further analyses of the age distribution of eyes with intraoperative complications found significantly more eyes within the age range of 1.5–3 years in the limbal group versus the pars plana group (Table 4).

All eyes had well-centered IOLs. Thirteen eyes had a three-piece IOL implanted in the sulcus. Besides the eight eyes with posterior capsule tears, the others had large posterior polar cataracts and a posterior capsulotomy of 5.5–6.0 mm was made. Placement of the IOL in the sulcus was safer and more stable.

### 3.3. Postoperative Complications

More eyes in the limbal group developed postoperative complications and required additional intraocular surgery though the difference was not significant (Table 3). These postoperative complications resulted in no known adverse sequelae.

## 4. Discussion

The optimal surgical technique for the removal of congenital cataracts in children using the microincision vitrectomy system remains unknown. The decision on whether to use the limbal or the pars plana approach requires balancing the visual benefits of the surgery against the risks associated with the procedure. During the follow-up period, there were no significant differences between the mean logMAR BCVA in eyes with congenital cataract that were operated on with either the limbal approach or the pars plana approach. However, in our hands, there were significantly more intraoperative complications and a trend of more postoperative complications using the limbal approach.

Children's delay in presentation for congenital cataract surgery is common in developing countries, making the visual rehabilitation more challenging for the ophthalmologists [16–21]. The mean delay between identification and surgery was 20.7 months to more than 8 years in different studies [16–20]. You et al. [19] reported a mean delay of presentation for surgery of 35.7 months in pediatric patients with congenital cataract in China. Lack of awareness, access to medical resources, and financial burdens deprive many children with congenital cataract of timely surgical treatment [20, 22]. Delayed surgical treatment is the major reason for severe visual impairment in pediatric patients with cataract in China [19]. Deep seated amblyopia occurred in these patients and aggressive amblyopic treatment after surgery is required. Although most cases surpassed the critical timing for surgery, performing cataract operation as soon as possible was the only effective option for these patients. Even in cases with years of delay for cataract treatment, visual improvement could still be achieved after cataract removal [18–20]. Postoperatively, appropriate optical correction and amblyopic treatment were initiated and efforts were made to encourage development of binocular function. Early detection of congenital cataract has been fulfilled by routine birth detection in developed countries, which makes it possible for ophthalmologists to perform cataract surgery at the proper time [23]. In China, more ophthalmologists are now making great efforts to avoid visual impairment resulting from late detection and surgery for congenital cataract. Establishment of a national screening system to ensure that all newborns are assessed for lens opacities, training of screening doctors and community medical practitioners, and education to caregivers of infants are of vital significance to enable children to access timely detection and treatment of congenital cataract [19, 21, 22].

To achieve good visual outcomes, a minimally invasive surgical procedure that accelerates postoperative recovery and enables immediate optical correction and amblyopic treatment is essential for visual rehabilitation in these children. The 25-gauge vitrectomy system has some limitations in the removal of less soft crystalline lenses or dense membranes and is only suitable for very young children. The 23-gauge vitrectomy system offers improvements in cutter efficiency and rigidity and is suitable for older children [5, 8]. Ahmadieh et al. [24] reported that no visual acuity, IOL position, or postoperative complications were found to be statistically significant between the limbal versus pars plana approach with primary capsulotomy, anterior vitrectomy, lensectomy, or posterior chamber IOL implantation in children using the 20-gauge vitrectomy system. Our results provide new insights into the comparisons between the two microincision vitrectomy approaches.

More complications were associated with disturbance and irritation to the iris in the limbal group. Manipulating the surgical instruments in the relatively small space of the shallow anterior chamber of children was a technical challenge when first using the technique in children. Inadvertent aspiration of iris tissue with the vitrectomy probe was common during the lensectomy and capsulotomy procedure, especially in eyes with small pupils. Ways to reduce iris prolapse and having the iris enter the cutting tip are as follows: (a) full dilation of the pupil; (b) deep level of general anesthesia using a depolarizing agent prior to making the first incision; (c) using intravenous mannitol if the globe is not sufficiently soft; (d) low flow on the infusion; (e) using a coaxial sleeve for irrigation around the cutting instrument; (f) biplanar incision construction; (g) using the incision as the fulcrum when repositioning the cutting tip. Using the incision to move the globe will stretch the incision, and prolapse will occur. Immediate release of the iris when iris aspiration occurs could help reduce the rate of iris injury. In cases of iris prolapse, injection of an ophthalmic viscosurgical device for repositioning and another suture for limbal incision are recommended to avoid iris incarceration. With surgical experience, no other case of iris incarceration occurred. The higher incidence of IOL pigmentation resulted from pigment dispersion and did not interfere with the visual axis.

The incidence of iris related complications was higher in our study compared with the previous reports using the microincision vitrectomy system for the management of congenital cataract [9–13]. The difference of intraoperative complications between the limbal and pars plana group is almost exclusively due to the problems associated with iris. However, this difference needs to be put into perspective, both short and long term. In order to compare the two surgical approaches more in detail, unlike other studies, we included the mild intraoperative complications that did not cause adverse sequelae. Of all the intraoperative complications, only one eye required additional surgery. The surgery to reposition the iris is simple and fast and has not been associated with additional complications. The four iris injuries were mild and did not interfere with the visual axis. In long-term perspective, at last follow-up, there were no significant differences in mean logMAR BCVA between the limbal and pars plana group. The intraoperative complications associated with the iris mostly occurred among the initial 20 eyes we performed using both approaches. With surgical experience, the incidence of iris related complication dropped dramatically and no other case requiring additional surgery occurred. If the iris problem was solved, the complication rate between the limbal and pars plana group may be not significantly different. There is reason to believe that as experience is gathered and technique is refined, the visual results and number of the complications between the two groups will become more even over the long term.

A more precise capsulotomy and more sufficient lensectomy and anterior vitrectomy were achieved using the pars plana approach. By inserting the 23-gauge vitrectomy cutter at the equator of the lens, the surgeon could turn the cutter upward to perform the anterior capsulotomy, forward and around to remove the lens materials, and downward to perform the posterior capsulotomy and anterior vitrectomy. Cutting was precise and sufficient and the chances of retained lens material resulting from insufficient removal of the central posterior capsule and the anterior vitreous were minimized [25].

The surgical procedure was simplified by enlargement of the main limbal incision for primary IOL implantation in the limbal approach. Surgically induced trauma was minimized and the learning curve of the limbal approach for the anterior segment surgeon was shorter than the pars plana approach. The more significantly intraoperative complications in the limbal group were mainly distributed in eyes within the age range of 1.5–3 years. Therefore, we recommend the limbal approach for children older than 3 years for a simplified procedure.

Finally, closure and water tightness of the incisions were easier using the limbal approach. The center of the limbal incision was relatively round because of the insertion of the microcannula and only one suture at this site was required. An additional suture was occasionally required to ensure the incision was watertight. For primary IOL implantation via the pars plana approach, another 2.6 mm limbal incision was made at the 12 o'clock position. Therefore, greater care should be taken to ensure that the three incisions are adequately closed and watertight at the end of the surgery. Repeated hydration of the incisions was usually needed. We did not observe wound leakage in any case. It is of vital significance to educate the children's caregivers to avoid any actions that might place pressure on the globe after surgery.

A limitation of this study is its retrospective aspect. A multicenter, prospective, randomized trial is needed to determine the optimal surgical approach for eyes with congenital cataract using the microincision vitrectomy system. However, gathering sufficient patients, controlling the variations in the surgical procedures, accomplishing the surgery at an earlier age, and conducting the procedure comparison in a time interval that would preclude advances in instrumentation or alterations in the procedure as improved techniques evolve are quite difficult in the short term. The perspective and information gained from the present study provide some basis for comparing the techniques in the management of congenital cataract with the microincision vitrectomy system.

In conclusion, the visual results were encouraging for the removal of congenital cataract using the microincision vitrectomy system and did not differ between the limbal and pars plana groups. The rates of intraoperative and postoperative complications were numerically higher in the limbal group but had no significant impact on long-term visual outcome. When operating on children with congenital cataract with primary IOL implantation using the microincision vitrectomy system, we recommend using the pars plana approach for a better safety margin. Also, greater care should be taken to make sure the three incisions are closed at the end of the surgery. The limbal approach should be reserved for children older than 3 years and caution exercised to minimize disturbance to the iris due to the potential risk of complications.

## Supplementary Material

Video 1 Surgical procedure of limbal approach
Two limbal incisions are made by a 23-gauge trocar with a microcannula. An infusion cannula and a vitrectomy cutter are introduced through the 4 or 8 o'clock and 12 o'clock incision, respectively. A central anterior capsulotomy of 5.0 mm diameter is created using the vitrector. Lens material is then removed. A posterior capsulotomy of 4.5 mm diameter is created followed by a limited anterior vitrectomy. The microcannula at the 12 o'clock incision is then removed. This incision is then enlarged to 2.6 mm. After the ophthalmic viscosurgical device (OVD) is injected, IOL is implanted into the capsular bag. The 12 o'clock limbal incision is closed with one 10-0 nylon suture and the limbal port incision is hydrated with BSS.
Video 2 Surgical procedure of pars plana approach
An infusion cannula is inserted through a limbal port incision to maintain the anterior chamber with BSS. A vitrectomy cutter is introduced through the pars plana incision, 2.5 mm posterior to the limbus. A central anterior capsulotomy of 5.0 mm diameter is created with the vitrector. Lensectomy is then performed. A posterior capsulotomy of 4.5 mm diameter is created with the vitrectomy cutter. Limited anterior vitrectomy is performed. The microcannula of the pars plana incision is removed without suturing. Another 2.6 mm limbal incision is made at the 12 o'clock position. After the OVD is injected, IOL is implanted into the capsular bag. The limbal incision is closed with one 10-0 nylon suture and the limbal port incision is hydrated with BSS.

## Figures and Tables

**Table 1:** Patient demographics.

Characteristics	Limbal (*n* = 40)	Pars plana (*n* = 41)	^a^ *P* value
Age at surgery, mean (range, months)	49 (18–72)	44 (18–72)	0.414
Category of age at surgery, *n* (eyes) (%)			
1.5 to 3 years	14 (35.0)	15 (36.6)	0.588/0.501^b^
3 to 5 years	19 (47.5)	15 (36.6)	
5 to 6 years	7 (17.5)	11 (26.9)	
Male/female	15/11	15/15	0.601
Unilateral, *n* (%)	11 (27.5)	16 (39.0)	0.347
Type of cataract, *n* (%)			
Total	13 (32.5)	16 (39.0)	0.082^b^
Fetal nuclear	10 (25.0)	11 (26.8)	
Lamellar	6 (15.0)	0 (0)	
Posterior polar	11 (27.5)	14 (34.1)	
Strabismus, *n* (%)	8 (20.0)	12 (29.3)	0.441
Nystagmus, *n* (%)	6 (15.0)	7 (17.1)	1.000
Axial length, (mm, range)	21.81 (19.48–24.77)	22.23 (19.03–24.66)	0.186
ECD, (mm^2^, range)	3128 (2693–3623)	3080 (2321–3577)	0.360
IOP (mmHg, range)	13.6 (9.1–19.0)	14.4 (10.1–20.3)	0.181
IOL position, *n* (%)			
Capsular bag	36 (90.0)	32 (78.0)	0.226
Sulcus	4 (10.0)	9 (22.0)	
IOL power, (D, range)	+22.2 (+13.0–+31.0)	+21.2 (+13.0–+30.0)	0.316
Follow-up time (months, range)	31 (24–48)	57 (36–77)	<0.001^a^

^a^ *P* < 0.05 = statistically significant. ^b^Pearson's chi^2^ test.

IOP = intraocular pressure; IOL = intraocular lens; D = diopter; ECD = endothelial cell density; *n* = number.

**Table 2:** Visual outcomes.

	Limbal (*n* = 40)	Pars plana (*n* = 41)	*P* value^a^
LogMAR BCVA			
Preoperative	1.15 (0.52–3.00)	1.17 (0.40–3.00)	0.904
Last follow-up visit	0.32 (0.00–1.30)	0.35 (0.00–1.30)	0.642
Refractive errors, (D)			
1 week postoperative	+1.75 (0.00–+3.00)	+2.18 (+0.50–+6.50)	0.171
Last follow-up visit	+0.71 (−2.00–+3.00)	−0.15 (−2.00–+2.75)	0.001^a^

BCVA = best-corrected visual acuity; logMAR = logarithm of the minimal angle of resolution.

^a^ *P* < 0.05 = statistically significant.

**Table 3:** Intraoperative and postoperative complications.

Characteristic	Limbal (*n* = 40)	Pars plana (*n* = 41)	*P* value^a^
*Intraoperative*			
Iris aspiration, *n* (%)	13 (32.5)	3 (7.3)	0.005^a^
Iris prolapse, *n* (%)	11 (27.5)	4 (9.8)	0.049^a^
Iris injury, *n* (%)	4 (10.0)	0	0.055
Tear of posterior capsule, *n* (%)	2 (5.0)	6 (14.6)	0.264
Lens fragment in vitreous, *n* (%)	1 (2.5)	0	0.494
*Eyes with at least 1 intraoperative complication*	17 (42.5)	8 (19.5)	0.032^a^

*Postoperative*			
Corneal clarity at 3 days or after, *n* (%)	40 (100)	41 (100)	1.000
Postoperative complications, *n* (%) (Time after surgery)			
IOL pigmentation	6 (15.0) (1 month)	2 (4.9) (1 month)	0.155
VAO required surgery	1 (2.5) (8 months)	1 (2.4) (2.5 years)	1.000
Iris incarceration in incision required surgery to reposition the iris	1 (2.5) (1 day)	0	0.494
IOL pupillary capture required surgery to reposition the IOL	1 (2.5) (1 week)	0	0.494
*Eyes with at least 1 postoperative complication*	7 (17.5)	3 (7.3)	0.194

D = diopter; CD = corneal endothelial cell density; IOL = intraocular lens; IOP = intraocular pressure; VAO = visual axis opacification.

^a^ *P* < 0.05 = statistically significant.

**Table 4:** Age distribution of eyes with intraoperative complications.

	Limbal (*n* = 40)	Pars plana (*n* = 41)	*P* value^a^
Category of age at surgery for intraoperative complications, *n* (%)			
1.5 to 3 years	10 (25.0)	3 (7.3)	0.009^a^
3 to 5 years	6 (15.0)	4 (9.8)	1.000
5 to 6 years	1 (2.5)	1 (2.4)	1.000
Category of age at surgery for postoperative complications, *n* (%)			
1.5 to 3 years	4 (10.0)	2 (4.9)	0.390
3 to 5 years	3 (7.5)	1 (2.4)	0.613
5 to 6 years	0	0	

^a^ *P* < 0.05 = statistically significant.
